# Notch3 is necessary for neuronal differentiation and maturation in the adult spinal cord

**DOI:** 10.1111/jcmm.12362

**Published:** 2014-08-28

**Authors:** Gabriel Rusanescu, Jianren Mao

**Affiliations:** 1 MGH Center for Translational Pain Research, Department of Anesthesia, Critical Care and Pain Medicine, Massachusetts General HospitalCharlestown, MA, USA

**Keywords:** Notch, chronic pain, spinal cord, stem cells, neuronal differentiation, neuronal progenitors, adult neurogenesis, calretinin, nociception, Notch3

## Abstract

Notch receptors are key regulators of nervous system development and promoters of neural stem cells renewal and proliferation. Defects in the expression of *Notch* genes result in severe, often lethal developmental abnormalities. Notch3 is generally thought to have a similar proliferative, anti-differentiation and gliogenic role to Notch1. However, in some cases, Notch3 has an opposite, pro-differentiation effect. Here, we show that Notch3 segregates from Notch1 and is transiently expressed in adult rat and mouse spinal cord neuron precursors and immature neurons. This suggests that during the differentiation of adult neural progenitor cells, Notch signalling may follow a modified version of the classical lateral inhibition model, involving the segregation of individual Notch receptors. Notch3 knockout mice, otherwise neurologically normal, are characterized by a reduced number of mature inhibitory interneurons and an increased number of highly excitable immature neurons in spinal cord laminae I–II. As a result, these mice have permanently lower nociceptive thresholds, similar to chronic pain. These results suggest that defective neuronal differentiation, for example as a result of reduced Notch3 expression or activation, may underlie human cases of intractable chronic pain, such as fibromyalgia and neuropathic pain.

## Introduction

Notch receptors initiate a signalling pathway that is essential for development and is conserved from invertebrates to mammals. The relative expression of Notch receptors, their Delta/Jagged ligands and of pro-neural bHLH transcription factors (*e.g*. Mash1/Ascl1, Ngn2) dictates stem cell fate during development through a lateral inhibition mechanism 1–9, which induces cellular divergence in a matrix of identical cells and guides somitogenesis and dorso-ventral patterning 10–14. Notch receptors promote stem cell renewal and proliferation 15 and are key developmental regulators, including the development of the nervous system. Defects in the expression or activation of Notch receptors result in severe, often lethal developmental abnormalities, which are especially evident in the nervous 16–19 and cardiovascular 20–23 systems. Notch signalling remains active in the adult nervous system 24, where it regulates adult neurogenesis 25,26.

The role of Notch3, one of four Notch receptors, is ambiguous: during brain development 17,27,28 and in some tumours 29–31 Notch3 appears to promote cell proliferation and gliogenesis similar to Notch1 32,33, the representative Notch receptor. In other cellular and developmental contexts, Notch3 has the opposite role, promoting cell differentiation 34–36. In adult goldfish, Notch3 is expressed in neurogenic regions and is up-regulated during retinal regeneration after injury 37. In adult rat spinal cord neurogenesis, Notch1 promotes cell proliferation 25, however the role of Notch3 is unknown.

Considering the role of Notch3 in promoting the differentiation of vascular smooth muscle cells *in vivo* 35 and of mesenchymal stem cells *in vitro* 36, we hypothesized that Notch3 may play a similar role in adult neural stem cells, despite its proliferative role in the embryonic nervous system. Neural stem cells and even adult neurogenesis have been previously observed in the adult mouse spinal cord under normal conditions in intact animals, although the generation of functional neurons through this process has been difficult to demonstrate 38,39. We first analysed the pattern of Notch3 expression in adult rat and mouse spinal cord, in comparison with neuronal and glial markers and other Notch receptors and ligands. This *in vivo* method was correlated with the *in vitro* analysis of the differentiation of Neuro-2a cells, neuroblastoma-derived cells that simulate neuronal differentiation. These data were compared with anatomical features specific to Notch3 knockout mouse spinal cord and with behavioural characteristics resulting from these features. Each of these methods supports our hypothesis that Notch3 is necessary for neuronal differentiation and maturation in the adult spinal cord.

## Materials and methods

### Animals and surgery

Two-month-old Sprague–Dawley (SD) male rats (Charles River Labs, Wilmington, MA, USA), Notch3 knockout (N3KO) and matching control B6129SF1/J mice (The Jackson Labs, Bar Harbor, ME, USA) were housed under standard conditions with free access to food and water. For behavioural experiments, mice were subjected to unilateral constriction injury of the right sciatic nerve (CCI) 40 under sodium pentobarbital anaesthesia (50 mg/kg, i.p.). Unilateral CCI was performed by placing four chromic gut ligatures around the right sciatic nerve. Mice not subjected to surgery (naïve) and mice subjected to sham surgery, without placing sciatic nerve ligatures, were used as controls. For immunofluorescence analysis, animals were killed by perfusion-fixation under anaesthesia. Experimental protocols were approved by the IACUC Committee at Massachusetts General Hospital.

### Immunofluorescence

Spinal cords were frozen in Tissue-Tek OCT and cryo-sectioned into transverse 35-μm-thick slices. Slices were permeabilized for 2 hrs (3% BSA and 0.2% Triton in PBS), then incubated overnight at 4°C with primary antibodies in 3% BSA/0.2% Triton/PBS, washed in PBS and incubated 1 hr with Cy3-or FITC-linked secondary antibodies (Jackson Immuno-Research, West Grove, PA, USA). Primary antibodies include Notch1, Notch2, Notch3, doublecortin, Mash1, neurogenin2, calretinin, Olig2, NogoA, βIII tubulin (TU20; Santa Cruz Biotechnology, Dallas, TX,USA), GFAP (BD Biosciences, San Jose, CA, USA), beta-actin (Abcam, Cambridge, MA, USA) and NeuN (Millipore, Billerica, MA, USA). Antibody specificity was attested by published reports and manufacturer's data, or tested by Western blot or by showing only partial overlap with chemical detection (EdU). Slices were mounted on slides and imaged using a Nikon 80 fluorescence microscope equipped with FITC and Cy3 filters. Bleed-through was minimized by dual scanning on two different FITC-Cy3 filter sets with slightly different band pass windows. 3D imaging was performed with a Keyence BZ-9000 microscope. Stereoscopic reconstruction and quantification were carried out using NIH ImageJ. Images shown are samples of at least 10 slices stained per antibody/per condition.

### EdU labelling

Two-month-old mice were injected daily for 7 days with 5-ethynyl-2-deoxyuridine (EdU, 4 mg/kg i.p., Jena Bioscience, Jena, Germany). On the 8th day, the mice were killed by perfusion-fixation with 4% paraformaldehyde/PBS under anaesthesia. Spinal cord slices were permeabilized 30 min. in 0.2% Triton/TBS, then incubated for 15 min. at room temperature with azide-fluorescein (Jena Bioscience) and 1 mM Cu^+^. The slices were washed and reprobed with primary antibodies, followed by Cy3-linked secondary antibodies. The slices were mounted on slides using glycerol-based mounting medium (Vectashield) and imaged by immunofluorescence.

### Western blots

Neuro-2a and derived cells, treated as shown, were lysed 15 min. in RIPA buffer containing protease inhibitors (aprotinin, leupeptin). Cleared lysates were assayed for total protein concentration with a colorimetric assay kit (Pierce, Rockford, IL, USA), were diluted 1:1 with Laemmli buffer to approximately 1 mg/ml, then boiled for 5 min. Samples containing equal amounts of protein were separated on 7–15% SDS-PAGE, then transferred to nitrocellulose membranes. Where indicated, protein immunoprecipitation was performed with Dynabeads Protein G (30 μl suspension/sample, Life Technologies, Grand Island, NY, USA), which were separated in a magnetic field and processed according to manufacturer's protocol. The blots were blocked in 5% BSA/TBST, incubated with primary antibodies as shown, washed 3× TBST, incubated with secondary antibodies, washed 3× TBST, exposed to ECL and imaged on a FluorChem M densitometric system.

### Cell culture and differentiation

Mouse neuroblastoma Neuro-2a cells grown in 10% FBS/DMEM were differentiated by replacing FBS with 1% horse serum (−FBS) for 5 days. Neuro2a-ΔN3 cell lines were derived from Neuro-2a by transfecting murine Notch3 intracellular domain (ICD, aa 1663-2318). Neuro2a-N3RNAi cell lines were derived from Neuro-2a by combined transfection of four plasmids expressing four different 29 bp Notch3 shRNAs (Origene Technologies, Rockville, MD, USA). Positive colonies were selected using puromycin, then analysed by Western blot for full-length Notch3 or ICD expression. For differentiation, cells with neurites longer than twice the cell body were counted as positives. Five random microscope fields per condition, each from an independent experiment, were analysed (*n* = 5).

### Behavioural tests

Mice were tested 1 day before CCI (Week 0) and retested weekly for mechanical nociceptive threshold (measured as hind-paw sensitivity to a series of von Frey filaments) and response latency to radiant heat (hind-paw withdrawal time in response to radiant heat stimulation) at the right hind paws, subjected to CCI. For mechanical pain threshold measurements, rats were placed in individual cages with wire mesh and accommodated for 30 min. Ascending Von Frey filaments (0.007–60 g) were applied five times each to hind paws’ palmar surfaces. The mechanical threshold was defined as the lowest force resulting in at least three withdrawals. For heat latency, a focused radiant light beam (Model 390; IITC Inc., Woodland Hills, CA, USA) was applied to the palmar surface four times at 10 min. intervals through a glass plate floor, at an intensity that resulted in a 12 sec. baseline withdrawal latency, with a 20 sec. cut-off. The average withdrawal time was defined as thermal withdrawal latency. The experimenter was blinded to experimental conditions.

### Statistical analysis

Immunofluorescence was quantified using NIH Image J and averaged for five random lumbar spinal cord slices per mouse, *n* = 5 mice. Neurite growth was analysed in five random microscope fields (*n* = *5*) from independent experiments. Western blots (ECL) were quantified on a FluorChem M densitometric system and analysed over five independent experiments, *n* = 5. Between-group comparison, for two different conditions (*i.e*. neuron numbers in WT *versus* N3KO mice, differentiation in Neuro2a *versus* Neuro2a-ΔN3), was performed by two-tailed *t*-tests. For the behavioural experiments shown, a sample size of *n* = 8 animals per condition was determined to be sufficient to pass the Shapiro–Wilk normality test and the equal variance test. Behavioural results were analysed using two-way repeated anova, considering two independent factors, treatment (N3KO *versus* WT, in Sham or CCI) and time (week = 0–4). This was followed by the Holm–Sidak *post hoc* test, consisting of multiple comparisons *versus* reference group. The reference group was assigned ‘Sham’ treatment at week 0 and the null hypothesis that there is no significant difference between the mean for each ‘treatment’ × ‘time’ pair and the reference group was tested against a significance level = 0.01. Testing was done every week in random order and position on the test surface. The significance of F-values for each anova analysis was tested against tabled F_critical_ values for alpha = 0.01. As a result of the non-linear sequence of von Frey filaments, some allodynia time-points, especially at high thresholds, did not pass the Shapiro–Wilk normality test and/or the equal variance test. However, Holm–Sidak pairwise multiple comparison tests confirmed statistically significant differences in means (*P* < 0.01) in each case.

## Results

### Notch3 is specifically expressed in neurons in the adult spinal cord

**Figure 1:**
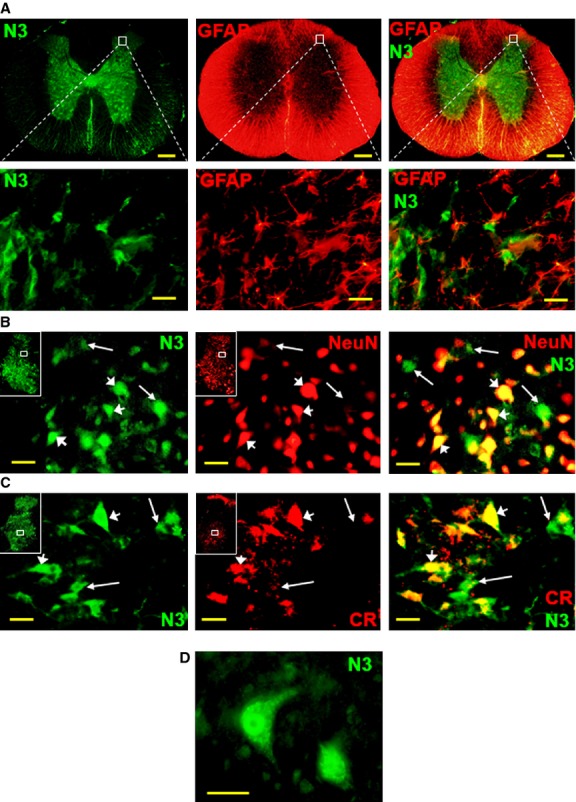
Immunofluorescence analysis of Notch3 expression in adult rat spinal cord. (**A**) Notch3 (N3) and astroglial marker GFAP are expressed in complementary spinal cord areas (grey matter *versus* white matter, respectively) and in non-overlapping cell populations (detail). (**B**) Spinal cord Notch3 expression partially co-localizes with neuronal marker NeuN (arrowheads), suggesting a gradual transition from Notch3+/NeuN-progenitors (arrows) to Notch3−/NeuN+ mature neurons (in red). Insets map the position of detail panels. (**C**) Notch3 is partially co-expressed with calretinin (CR), a marker for a sub-set of inhibitory GABAergic interneurons. The arrows indicate cells at various stages of differentiation: Notch3+/CR− (arrows), Notch3+/CR+ (arrowheads). (**D**) Notch3 (N3) is preferentially expressed in the nuclei of cells with neuroblast phenotype. Scale bars: 200 μm (**A**), 20 μm (A-details, **B**–**D**).

To investigate the role of Notch3 in the adult spinal cord, we first analysed its expression pattern. Spinal cord sections from 2-month-old SD rats were analysed by immunofluorescence for Notch3 expression, in parallel with neuronal markers NeuN, calretinin (CR) and astroglial marker GFAP. Notch3 is almost exclusively expressed in the grey matter, complementary to GFAP expression (Fig.1A). In the upper dorsal horn layers, which appear to express both Notch3 and GFAP, Notch3 is expressed in cells clearly distinct from astroglia and does not co-localize with GFAP (Fig.1A, details). In contrast, Notch3 expression partially overlaps with NeuN staining (Fig.1B). The coexistence of Notch3+/NeuN− (18 ± 3% frequency in the dorsal horn, *n* = 5 rats), Notch3+/NeuN+ (48 ± 5%) and Notch3−/NeuN+ (34 ± 4%) cells suggests that Notch3 is transiently expressed in neuron precursors and/or immature neurons at a stage that precedes full neuronal maturation indicated by NeuN expression. The fraction of Notch3+/NeuN+ cells includes a continuum of Notch3/NeuN expression ratio. Notch3 is expressed in neurons of all sizes, including small neurons in the upper dorsal horn, as well as in some large ventral horn motor neurons (Fig. S1). A similar partial overlap occurs between Notch3 and CR, a GABAergic neuron marker [Fig.1C, Notch3+/CR− (26 ± 4%, *n* = 5), Notch3+/CR+ (59 ± 6%), Notch3-/CR+ (15 ± 2%)]. Such incompletely differentiated neurons may result from adult neurogenesis 38,39 or may be the result of impaired neuronal maturation during development. The co-expression of Notch3 with two independent neuronal markers suggests that Notch3 is expressed in the adult in cells with neuronal phenotype instead of glial precursors as during development 27,28. Some Notch3-expressing cells, with a neuroblast phenotype, clearly show a nuclear accumulation of Notch3 (Fig.1D), including in cells that co-express NeuN or CR, reflecting Notch3 activation, cleavage and migration of its ICD to the nucleus 8. The nuclear expression of Notch3 and its co-localization with NeuN were confirmed by 3D analysis (Fig. S2). Similarly to the absence of Notch3 expression in astroglia, Notch3 does not co-localize with oligodendrocyte markers Olig2 or NogoA (Fig. S3).

**Figure 2:**
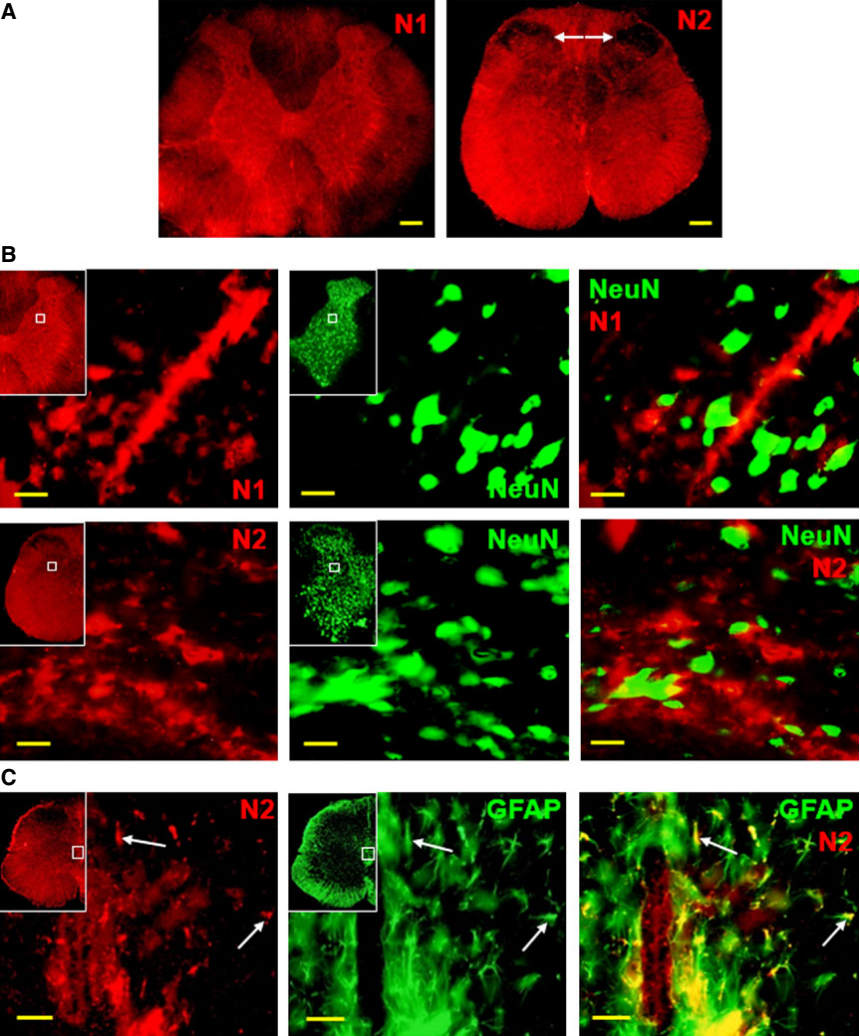
Comparative expression of Notch receptors in rat spinal cord. (**A**) Notch1 (N1, Cy3) and Notch2 (N2, Cy3) are ubiquitously expressed in rat spinal cord. Notch2 expression is lowest in laminae I–II (arrows). (**B**) Notch1 and Notch2 are not expressed in neurons (NeuN+ staining, FITC). Insets map the position of detail panels. (**C**) Notch2 is expressed in a fraction of sub-ependymal zone astrocytes (GFAP staining, FITC, arrows). Scale bars: 200 μm (**A**), 20 μm (**B**), 50 μm (**C**).

In contrast to Notch3, Notch1 and Notch2 are expressed throughout the spinal cord (Fig.2A), and Notch2 is specifically excluded from the upper dorsal horn layers (arrows). Neither Notch1 nor Notch2 co-localize with neuronal marker NeuN (Fig.2B), while Notch2 is expressed in a sub-set of GFAP-positive cells in the sub-ependymal cell layer, suggesting it may be implicated in the generation of radial glial cells 32,33 (Fig.2C). These data indicate that Notch3 could play a distinct role in adult spinal cord neuronal differentiation, in contrast with Notch1-2.

### Notch3 is expressed in adult neuron progenitor cells

**Figure 3:**
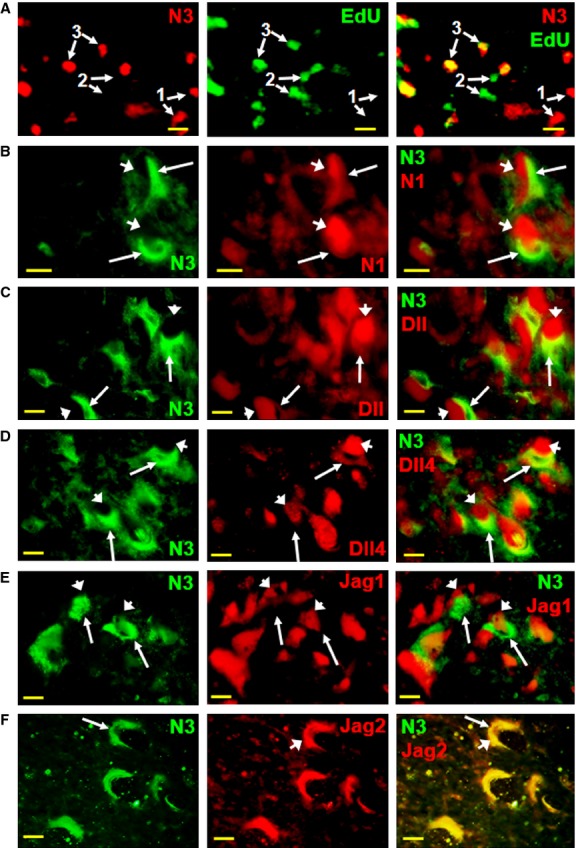
Notch3 is expressed in adult mouse spinal cord neural progenitor cells. (**A**) Notch3 expression (N3) partially overlaps with cell proliferation marker EdU. 1: Notch3+/EdU−, cells that stopped proliferating prior to EdU treatment; 2: Notch3−/EdU+, proliferating cells; 3: Notch3+/EdU+, proliferating cells that have committed to a neuronal fate. (**B**) Notch3 (N3, green, arrows) and Notch1 (N1, red, arrowheads) are expressed in pairs of juxtaposed cells. (**C**–**E**) Notch3-expressing cells (N3, green, FITC, arrows) appear paired with cells expressing Notch ligands Delta (Dll, **C**), Delta 4 (Dll4, **D**) and Jagged 1 (Jag1, **E**) (red, Cy3, arrowheads), potentially as a result of a lateral inhibition mechanism that diverges neighbouring cells. (**F**) Notch3 is co-expressed with ligand Jagged 2 (Jag2, arrowheads), in contrast to the other Notch ligands; scale bars: 10 μm.

**Figure 4:**
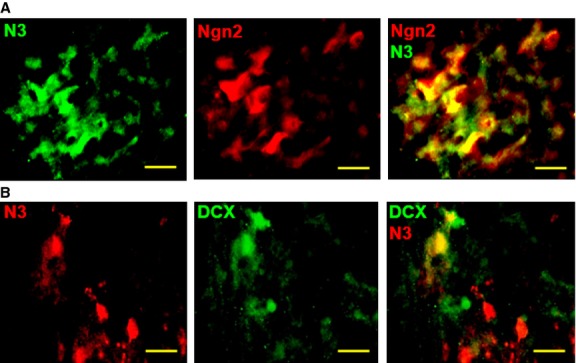
Notch3 is co-expressed with neuronal progenitor markers in mouse spinal cord. (**A**) Notch3 is co-expressed with pro-neural marker Ngn2. (**B**) Notch3 is partially co-expressed with neurogenesis marker DCX; scale bars: 20 μm.

Notch signalling has been previously implicated in the initial stages of embryonic stem cell differentiation, while our data suggest that Notch3 is expressed during the final stages of adult neuronal differentiation. Therefore, we examined whether Notch3 is also expressed in adult neural progenitor cells (NPCs), which have been shown to be normally present in the adult spinal cord 38,39. Subsequent observations were made in mouse spinal cord, which shows a similar neuron-specific Notch3 expression pattern to rat (Fig. S4). We traced newly generated proliferating cells by labelling with 5-ethynyl-2-deoxyuridine (EdU), a thymidine and BrdU analogue, which is incorporated during DNA replication 41. EdU was detected chemically at room temperature by azide-alkyne Huisgen cycloaddition (‘click’ chemistry) with an azide-linked fluorescent dye, which allows subsequent efficient co-staining with primary antibodies. Cells co-stained for EdU and Notch3 (Fig.3A) indicate proliferating cells destined to a neuronal fate, as suggested by Notch3 expression. The presence of newly generated adult NPCs in the spinal cord, in the same time frame as Notch3/EdU co-localization, can be demonstrated by the co-localization of EdU and synapsin I, a well-known neuronal marker that is expressed from the earliest stages of neuronal differentiation to the mature neuron stage 42 (Fig. S5). The differentiation of adult neural stem cells may recapitulate many stages of the embryonic development, in which Notch plays a key role in deciding cell fate through a lateral inhibition mechanism between Notch receptors and ligands. Therefore, we compared the expression of Notch3 with that of Notch1 and Notch ligands Delta and Jagged. Interestingly, Notch1 and Notch3 are expressed in the spinal cord grey matter in an exclusively complementary pattern, in pairs of adjacent cells (Fig.3B), suggesting that a lateral inhibition process also occurs between individual Notch receptors. This reciprocal exclusion of Notch1 *versus* Notch3 expression in neighbouring cells is in agreement with their divergent spinal cord expression, *e.g*. the neuron-specific expression of Notch3 *versus* non-neuronal Notch1 expression (compare Fig.1 *versus* Fig.2). This indicates that Notch3 induces different cell lineages than Notch1 during adult NPC differentiation. A similar complementary expression pattern occurs between Notch3 and Notch ligands Delta (Dll), Delta 4 (Dll4) and Jagged 1 (Jag1) (Fig.3C–E), suggesting that these ligands are expressed in adjacent non-neuronal cells. In contrast, Jagged 2 (Jag2) expression overlaps with Notch3 (Fig.3F). A similar pattern of Delta and Jagged expression is present at a macroscopic level, in whole spinal cord sections. Dll, Dll4 and Jag1 are preferentially expressed in the white matter, complementary to Notch3 expression, while Jag2 is preferentially expressed in the grey matter, matching Notch3 expression (Fig. S6). This Delta/Jagged expression pattern in the adult is different from the embryonic development, where Dll and Jag1 are expressed in cells with neuronal fate. Therefore, in contrast to the segregation of Notch receptors *versus* ligands as a group, which defines the classical lateral inhibition model during embryonic development, adult neuronal differentiation may follow a slightly different mechanism, involving the segregation of individual Notch receptors (*e.g*. Notch1 *versus* Notch3) and ligands (*e.g*. Jag1 *versus* Jag2). To further analyse the role of Notch3 in NPC differentiation, we investigated the co-expression of Notch3 with sequential NPC markers neurogenin2 (Ngn2) 43 and doublecortin (DCX) 44. Notch3 is partially co-expressed with both markers (Fig.4), indicating a continuum of Notch3 expression in NPCs.

### Notch3 is necessary and sufficient for neuronal differentiation

**Figure 5:**
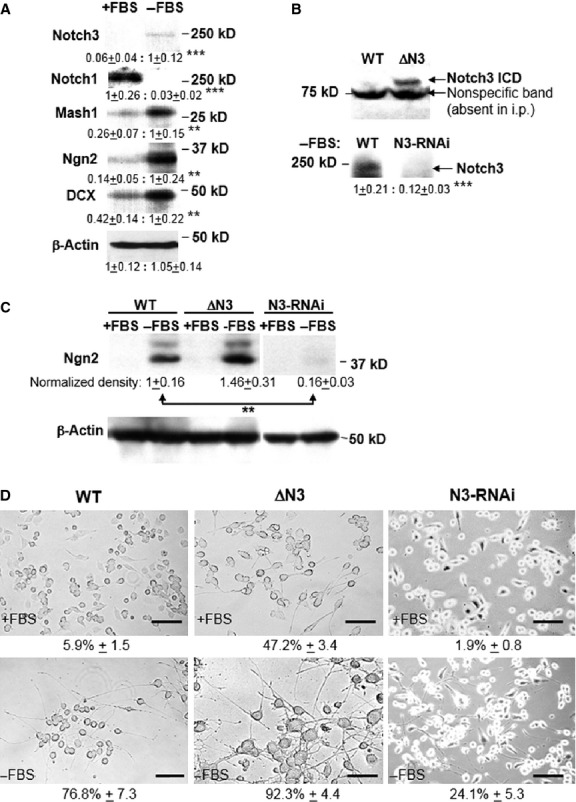
Notch3 is necessary for the expression of neurogenic markers and for the neuronal differentiation of Neuro-2a cells. (**A**) Expression of Notch3, Notch1, Mash1, Ngn2 and DCX in mouse Neuro-2a cells cultured in 10% FBS (+FBS) or in 1% HS (−FBS) for 5 days. β-actin indicates relative sample concentration. Data indicate the normalized concentration of each protein ±SEM and the statistical significance of their change after FBS withdrawal (*n* = 5). (**B**) Notch3 expression in Neuro-2a-derived ΔN3 (transfected Notch3 intracellular domain ICD is shown) and N3-RNAi cell lines. Notch3 expression in WT/N3-RNAi cells is shown under differentiating conditions (5 days in 1% HS medium), as Neuro-2a cells grown in FBS lack detectable full-length Notch3. Samples shown for WT/N3-RNAi cells represent Notch3 immunoprecipitates. (**C**) Notch3 knock-down (in N3-RNAi cells) prevents the expression of pro-neural marker Ngn2 under differentiating conditions (−FBS). (**D**) Notch3-dependent regulation of Neuro-2a cells differentiation. Activated Notch3 ICD (ΔN3 cells) promotes, while Notch3 knock-down (N3-RNAi cells) prevents neurite outgrowth in Neuro-2a cells, under both proliferating (+FBS) and differentiating (−FBS) conditions. Data indicate mean % cells with neurites ±SEM; scale bars: 200 μm.

The *in vivo* data presented so far suggest that Notch3 is associated with neuronal differentiation, from the initial divergence of the neuronal lineage from adult neural stem cells, to the final stages of neuronal differentiation and maturation. We tested this hypothesis *in vitro*, in mouse neuroblastoma Neuro-2a cells, a cellular model with neuronal progenitor characteristics that undergoes neuronal differentiation under serum deprivation. A 5-day serum deprivation of Neuro-2a cells results in increased Notch3 protein expression and a simultaneous decrease in Notch1 expression (Fig.5A, −FBS). The difference between the large decrease in Notch1 and the modest increase in Notch3 expression during differentiation may result from a reduced cell–cell contact between differentiated cells relative to the extensive contact within proliferating cell clusters, which is likely to affect Notch expression, or may be simply because of different antibody affinities. The decline in Notch1 expression during differentiation is in agreement with its known proliferative role. In contrast, the increased Notch3 expression, which parallels the increased expression of pro-neural markers Mash1, Ngn2 and neurogenic marker DCX, suggests that Notch3 would have a predominantly pro-differentiation role. To test this hypothesis, we generated stable Neuro-2a cell lines that express either the Notch3 ICD, which mimics Notch3 cleavage and activation (ΔN3 cells), or four different Notch3 shRNAs that induce Notch3 down-regulation by ∼90% (N3-RNAi cells) (Fig.5B). We used these cell lines to analyse the effect of Notch3 on the expression of pro-neural transcription factor Ngn2 (Fig.5C). Relative to the induction of Ngn2 expression observed in wild-type Neuro-2a cells after serum deprivation (WT, 1 ± 0.16, mean normalized band density ± SEM), Ngn2 expression was marginally enhanced in ΔN3 cells (1.46 ± 0.31, two-tailed *t*-test, *P* = 0.24, *n* = 5 independent experiments), and almost completely blocked in N3-RNAi cells (0.16 ± 0.028, *P* = 0.008 < **P = 0.01), supporting Notch3 implication in neuronal differentiation. Subsequently, these cell lines were tested in a differentiation assay, as measured by the proportion of cells with neurites. As expected, WT Neuro-2a cells maintained an undifferentiated state when grown in 10% foetal bovine serum (Fig.5D, WT+FBS, 5.9 ± 1.5, mean % cells with neurites ± SEM, *n* = 5 independent experiments) and began to show the hallmarks of neuronal differentiation, including neurite growth, within 5 days after FBS deprivation (Fig.5D, WT-FBS, 76.8 ± 7.3). In ΔN3 cells, neurite growth was enhanced even under normal serum conditions (Fig.5D, ΔN3 + FBS, 47.2 ± 3.4, two-tailed *t*-test relative to WT + FBS, *P* = 2 × 10^−6^ < ***P = 0.001). Under FBS deprivation, ΔN3 cells developed large cell bodies and an extensive neurite network (ΔN3 − FBS, 92.3 ± 4.4, *P* = 0.0008 < ***P = 0.001 relative to WT − FBS). In contrast, N3-RNAi cells, smaller in size and less adhesive than WT, showed almost no neurites when grown with FBS (N3RNAi + FBS, 1.9 ± 0.8, *P* = 0.005 < **P = 0.01 relative to WT + FBS), and developed few, shorter neurites even under FBS deprivation (N3RNAi − FBS, 24.1 ± 5.3, *P* = 7 × 10^−5^ < ***P = 0.001 relative to WT − FBS). These results complement the immunofluorescence observations, strongly supporting a key role for Notch3 in promoting neuronal differentiation, in contrast to Notch1.

### Notch3 knockout mice show impaired neuronal maturation

**Figure 6:**
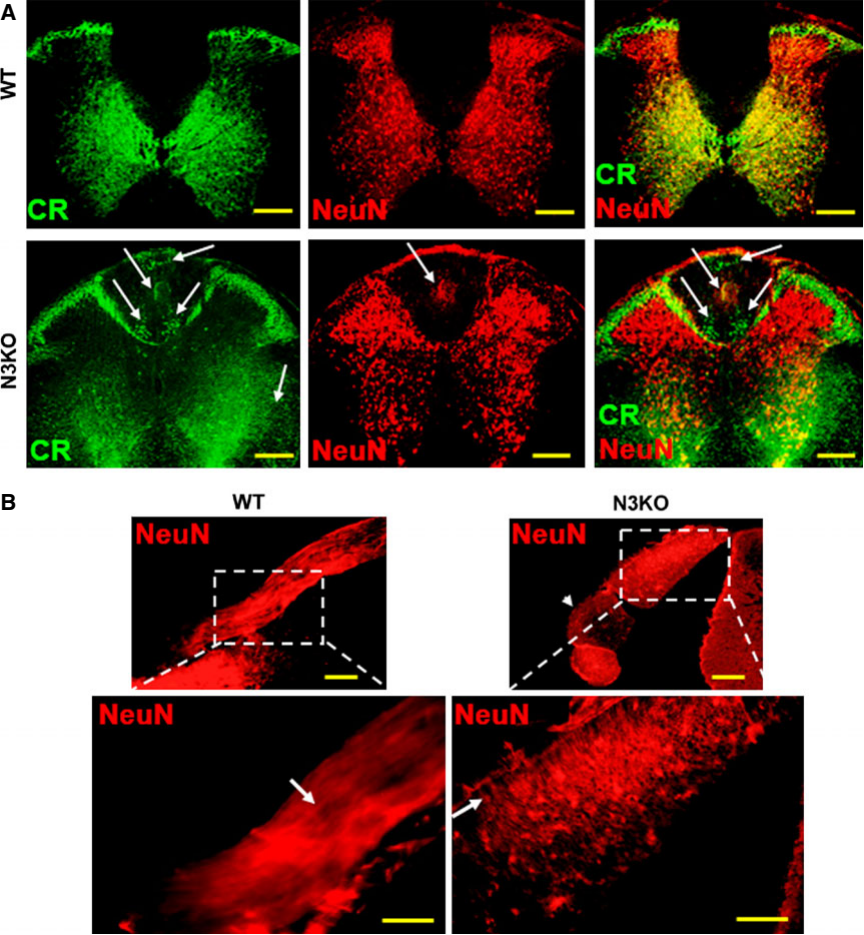
N3KO mice present spinal cord anatomical defects relative to WT. (**A**) Impaired maturation allows neurons to infiltrate into the white matter (CR+ cells, arrows) and to form abnormally located neuronal nuclei within the dorsal column (NeuN staining, arrow). While in WT mice CR+ cells are located centrally around the spinal cord central canal, in N3KO mice CR+ cells are located towards the periphery of the grey matter and penetrating into the surrounding white matter. (**B**) Defective neuronal maturation in N3KO mice allows immature neurons to migrate into the spinal nerves and dorsal root ganglia. Spinal nerves appear shorter and wider than in the WT. Several dorsal root ganglia are enlarged and grouped together in the proximity of the spinal cord (arrowhead). These ganglia include axons that run transversally to the direction of the spinal nerve sensory axons shown in WT (details, arrows). Scale bars: 200 μm (**A**), 100 μm (**B**), 50 μm (B-details).

**Figure 7:**
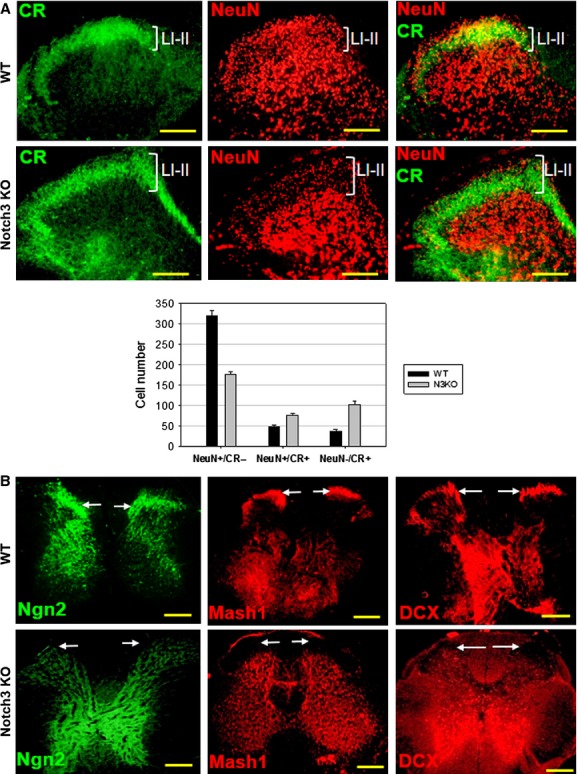
Notch3KO mice express lower levels of pro-neural and neuronal markers in laminae I–II. (**A**) Upper spinal cord dorsal horn, showing reduced mature neuron staining (NeuN) and increased staining for immature CR+/NeuN− neurons in N3KO mouse laminae I–II, relative to WT (LI–II). The increased number of CR+ neurons may create an outward pressure that could explain the unusual rectangular shape of the CR+ layer. Data indicate mean number of cells ±SD,*n* = 5. (**B**) Notch3 KO mice lack the expression of neural progenitor markers Mash1, Ngn2 and DCX in laminae I–II (arrows), which is present in WT mice. Scale bars: 100 μm (**A**), 200 μm (**B**).

To analyse the physiological implications of Notch3 expression, we examined commercially available Notch3 knockout mice (N3KO) 45 both anatomically and behaviourally. These mice do not show any overt signs of neurological deficits and to our knowledge no neurological abnormality has been reported 45. However, we found that these mice have a number of spinal cord anatomical differences relative to the WT mice. As a general observation, spinal cord dorsal horns are shorter in N3KO mice relative to WT (Fig.6A, Fig. S7). With the exception of lamina I/II, calretinin-positive (CR^+^) cells are located in WT mice towards the central grey matter, surrounding the spinal cord central canal, while in N3KO mice, CR^+^ cells are located towards the peripheral grey matter and even penetrating into the surrounding white matter (Fig.6A, arrows). This evidence suggests an impairment in neuronal maturation, which coincides with the observations during N3-RNAi cells differentiation. This impaired maturation would allow neurons to migrate further away from the central canal, into the spinal cord dorsal column and even into the spinal nerves (Fig.6B, Fig. S8), where they may establish additional neural networks. Spinal nerves and dorsal root ganglia appear enlarged in N3KO mice, likely because of a large number of immature neurons migrating from the spinal cord. N3KO mice have 3–4 adjacent dorsal root ganglia, with variable neuron densities, repositioned along each spinal nerve in close proximity to the spinal cord (Fig.6B, arrowhead, and Fig. S8). Another key anatomical feature in N3KO mice is a reduced level of NeuN staining in dorsal horn laminae I/II relative to WT (Fig.7A and Fig. S9). These spinal cord layers contain 45% fewer mature NeuN^+^/CR− neurons (176.2 ± 5.8, mean cell number ± SEM) relative to matching control (WT) mice (320.3 ± 11.5, two-tailed *t*-test, *P* = 4 × 10^−6^ < ***P = 0.001). In addition, NeuN+ neurons that are still present in N3KO mice in laminae I/II show weaker NeuN staining, suggesting that these cells express lower NeuN levels. In contrast, N3KO mice have a higher density of NeuN+ cells relative to WT in laminae III–IV, matched by a higher density of nuclear DAPI staining demonstrating more cells (Fig. S7, details).

In contrast to lower NeuN expression, laminae I/II express in N3KO mice significantly higher levels of calretinin. In addition to being a GABAergic neuron marker, calretinin expression may also indicate the presence of immature neurons 46–48. We found that N3KO mice had 274.5% more NeuN−/CR^+^ cells in laminae I/II (103.2 ± 7.1, mean cell number ± SEM, two-tailed *t*-test, *P* = 6 × 10^−7^ < ***P = 0.001) relative to WT (37.6 ± 3.8) and 57% more NeuN+/CR+ cells (76.6 ± 4.8 *versus* 48.8 ± 3.6 in WT, *P* = 2 × 10^−6^ < ***P = 0.001) (Fig.7A). As CR is a neuronal marker, the absence or reduction in NeuN expression in many laminae I–II CR^+^ cells suggests that these neurons are at an incomplete maturation stage. In agreement with the data shown in Figure5C for N3-RNAi cells, the impaired neuronal maturation observed in N3KO mice correlates with the absence of NPC markers Mash1, Ngn2 and DCX in laminae I/II, which are normally expressed in WT mice (Fig.7B).

### Notch3 knockout mice demonstrate chronically elevated nociception

**Figure 8:**
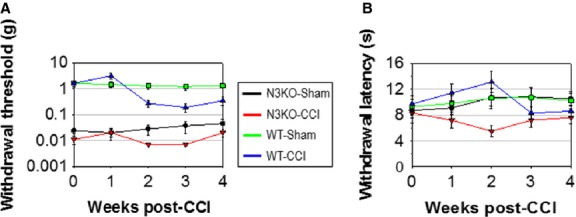
N3KO mice have chronically increased nociception. (**A**) N3KO mice display lower nociceptive threshold to mechanical stimuli. Basal nociceptive threshold to mechanical stimuli is almost two orders of magnitude lower in N3KO-Sham *versus*WT-Sham. A similar nociceptive threshold difference persists between N3KO and WT mice during post-CCI allodynia, when thresholds are shifted lower in both strains (N3KO-CCI *versus*WT-CCI). (**B**) N3KO mice show increased sensitivity (shorter latency) to noxious thermal stimuli. Basal response time to noxious heat is similar in N3KO and WT mice (N3KO-Sham *versus*WT-Sham), however, post-CCI hyperalgesia shows a shorter response time in N3KO mice (N3KO-CCI *versus*WT-CCI). Nociceptive responses in control naïve mice are similar to Sham and have been omitted. Data indicate mean nociceptive threshold ±SEM,*n* = 8. As a result of the semi-log scale, +/− error bar pairs may appear asymmetrical.

Spinal cord laminae I–II mediate the transmission of nociceptive signals from the peripheral nervous system to the brain. Sensory myelinated Aδ and non-myelinated C fibres provide input to laminae I–II neurons, many of which are inhibitory GABAergic CR+ interneurons. The anatomical abnormality observed in laminae I–II suggested that N3KO mice could have an altered behavioural response to nociception. This hypothesis was tested by comparing the nociceptive thresholds to mechanical and thermal stimulation in N3KO *versus* WT mice. We found that in N3KO mice, the pain threshold to mechanical stimuli is approximately two orders of magnitude lower than in wild-type mice (Fig.8A, N3KO-Sham *versus* WT-Sham, two-way repeated anova,*n* = 8 mice per condition, F = 479 > Fcritical = 21.2, ***P < 0.001), while heat sensitivity is similar to wild-type (Fig.8B, N3KO-Sham *versus* WT-Sham, *n* = 8, F = 0.79 < Fcritical = 21.2, *P* = 0.38). The nociceptive susceptibility of N3KO *versus* WT mice was further analysed in a commonly used rodent model of chronic nociception, the unilateral chronic constriction injury of the sciatic nerve (CCI) 40. WT mice subjected to CCI display mechanical allodynia (a decrease in mechanical nociceptive threshold to non-noxious stimuli; Fig.8A, WT-CCI *versus* WT-Sham) and heat hyperalgesia (increased heat sensitivity measured as the response latency to radiant heat stimulation; Fig.8B, WT-CCI *versus* WT-Sham). After CCI, N3KO mice display a significantly lower nociceptive threshold to mechanical stimulation (Fig.8A, N3KO-CCI *versus* WT-CCI, F = 129 > Fcritical = 21.2, ***P < 0.001) and shorter withdrawal latency to noxious heat stimulation relative to wild-type mice (Fig.8B, N3KO-CCI *versus* WT-CCI, F = 341 > Fcritical = 21.2, ***P < 0.001). This increased nociceptive susceptibility in N3KO mice is consistent with the larger number of immature CR+/NeuN− neurons in laminae I–II, in which GABA has an excitatory, depolarizing effect 49, and with fewer mature, inhibitory NeuN+ neurons. The result of the defective neuronal maturation in N3KO mice is therefore a high level of sensory excitability. These findings further support the notion that Notch3 is required for neuronal differentiation and maturation in the adult spinal cord.

## Discussion

In this study, we have investigated the cellular and functional roles of Notch3 in the adult spinal cord. During nervous system development, Notch3 has a similar proliferative action with Notch1, however its role in the adult nervous system is unknown. Previous reports have suggested that, in contrast to Notch1-2, Notch3 can have a pro-differentiation role in a variety of cellular contexts, including pancreas development 34, smooth muscle cell maturation 35 and mesenchymal stem cell calcification 36. This may result from the fact that Notch3, a weaker inducer of downstream *HES* genes, can antagonize Notch1 by competing for the *HES* promoters 50. Here, we have investigated Notch3 function in the adult rodent spinal cord using cellular, molecular, anatomical and behavioural approaches. Our results indicate that Notch3 has a divergent role from Notch1 beginning with the earliest stages of adult neural stem cell differentiation. We found, by comparison with the expression of sequential markers of neurogenesis, that there is a continuum of Notch3 expression from early to late neural progenitors and immature neurons, including in some NeuN+ cells, which are thought to represent mature neurons.

### Notch3-dependent neuronal differentiation deviates from the classical lateral inhibition model

In the classical lateral inhibition model of embryonic neurogenesis, Notch receptors are thought to segregate as a group from their Delta and Jagged ligands expressed by neighbouring cells, initiating the divergence of the neuronal lineage, cells that exclusively express ligands but not Notch receptors. Our observations in adult rodent spinal cord indicate that Notch3 and Notch1 segregate in adjacent cell pairs, and have diverging expression patterns. Notch3 is expressed exclusively in the grey matter, is up-regulated during neuronal differentiation and co-localizes with pro-neural and neuronal markers, while Notch1 is expressed throughout the spinal cord, but not in neurons, and is down-regulated during neuronal differentiation. The pairs of Notch1-and Notch3-expressing cells suggest that they may have been generated from the same progenitor cell, either through asymmetric division or through asymmetric differentiation after division. This suggests a variation from the standard lateral inhibition paradigm, where Notch1 and Notch3 have similar proliferative, gliogenic roles. Additional deviations from the standard lateral inhibition model include the predominantly white matter expression of Delta and Jag1 and their exclusion from neuronal lineage cells, and the co-expression of Notch3 receptor and Jag2 ligand. In the classical lateral inhibition model, ligand expression inhibits the expression of Notch receptors in the same cell, therefore the co-localization of Notch3 and Jag2 in the adult represents an exception from this model. The initial divergent expression of Notch1 and Notch3 appears to be essential for the progression of neuronal differentiation, as Notch3 goes on to co-express with early and late neuronal differentiation markers, in contrast to Notch1/2. These differences may result from different cellular environments in the adult *versus* embryo. While the early embryo involves a layer of identical stem cells in contact, adult spinal cord stem cells are isolated among multiple types of already differentiated cells, which may assist the Notch1/Notch3 divergence through a variety of signalling molecules expressed or secreted by other neighbouring cells. It remains to be determined whether this variation from the standard lateral inhibition model is specific for the spinal cord or for adult neuronal differentiation, or it is a general characteristic of Notch3 signalling.

### Notch3 regulates the differentiation of spinal cord laminae I/II neurons

Although Notch3/Notch1 segregation in the initial stages of NPC differentiation would suggest that Notch3 plays a key role in neuronal differentiation, we detected only limited nervous system abnormalities in N3KO mice, in particular in the spinal cord laminae I/II, although more subtle changes cannot be excluded. This abnormality is associated with the altered expression of transcription factors Mash1 and Ngn2 and of neurogenesis marker DCX*,* and is validated by a corresponding sensory behaviour. Changes in pro-neural gene expression may lead to defects in the expression of homeodomain genes such as *Lbx1,* which specify the identity of superficial dorsal horn interneurons involved in nociceptive signalling 51–53. It is interesting that Mash1 and low Ngn2 levels continue to be expressed in laminae I/II in wild-type adult mice, suggesting that neuronal differentiation is normally delayed or incomplete in these layers. Surprisingly, despite the lack of Mash1 and Ngn2 expression in the superficial dorsal horn layers of N3KO mice, neuronal differentiation in laminae I/II still progresses to the stage of immature, CR-expressing neurons. This observation, together with the limited extent of nervous system defects in N3KO mice, suggests that Notch3 function is partially redundant with other signalling molecules, possibly Jag2, which is co-expressed with Notch3 and may also contribute to the divergence of the neuronal lineage. The enhanced differentiation of Neuro2a-ΔN3 cells in the presence of serum even in the absence of Ngn2 expression suggests that Ngn2 is not absolutely required for neuronal differentiation 54,55 and that Notch3 may induce alternate pro-neural transcription factors. Furthermore, the roles of Mash1 and Ngn2 may be partially compensated by other similar pro-neural transcription factors that could be expressed independently of Notch3.

The data shown here are insufficient to evaluate whether the CR+ laminae I–II immature neurons observed in the adult N3KO mouse spinal cord have originated in the adult or are remnant from the embryonic development. CR has been shown to be transiently expressed in neurons during both nervous system development 46 as well as during adult neurogenesis 47,48. The nuclear expression of CR, in agreement with previous observations 56, is consistent with a key role of CR in nuclear calcium regulation and gene expression during neuronal differentiation 57. Our observation that Notch3 is co-expressed with CR in a number of spinal cord cells, including in their nuclei, supports the notion that Notch3 is expressed in immature neurons, suggesting that Notch3 plays different, even opposite, roles in the adult *versus* embryonic nervous systems.

### Notch3-dependent neuronal maturation in the adult spinal cord modulates pain behaviour

The anatomical and behavioural abnormalities exhibited by N3KO mice may be relevant for human chronic pain. In immature, CR+/NeuN− GABAergic neurons GABA has a depolarizing rather than hyperpolarizing effect because of the expression of the NKCC1 transporter, which maintains a high intracellular Cl^−^ concentration 58,59. This would result in N3KO mice in significantly increased excitability in laminae I–II that contains more such neurons relative to wild-type. Increased spinal cord excitability has been associated in humans with chronic pain, including neuropathic pain and fibromyalgia 60,61. We expect that future electrophysiological studies will validate our multiple lines of evidence indicating increased excitability of lamina I–II neurons in N3KO mice. In addition, the larger and more numerous dorsal horn ganglia and the neurons inserted inside the spinal nerve, present in N3KO mice as a result of impaired neuronal maturation, may also contribute to the increased nociception. We cannot exclude that changes in other central nervous system areas involved in pain transmission and processing could contribute to the increased nociception observed in N3KO mice. However, we were unable to detect in the N3KO mouse brain any macroscopic changes similar to those observed in the spinal cord. In addition, the anatomical changes in N3KO mouse spinal cord are sufficient to explain the observed behavioural alterations.

As a result of the obvious difficulty of studying in humans the cellular events underlying chronic pain, several animal models that simulate chronic pain have been generated, such as CCI for neuropathic pain 40 or reserpine induced myalgia for fibromyalgia 62. However in these animal models, the increased sensitivity to nociception, induced by mechanical or chemical injury, subsides after 2 months or less, once the underlying injury has healed, unlike humans, where chronic pain can last with no apparent cause for years after the healing of underlying pathology. Therefore, N3KO mice, which show lifelong increase in nociception, may be a more appropriate model for human chronic pain. In addition, these mice show daytime hyperactivity similar to the insomnia, restless leg syndrome and anxiety associated with fibromyalgia.

While in the case of chronic neuropathic pain no genetic contribution has been yet demonstrated, fibromyalgia has a clear, yet unresolved, genetic component 63, which has been also linked to coincident cancer and cardiovascular disease susceptibility 64, both of which can result from abnormal Notch signalling. Fibromyalgia has been genetically linked to the HLA region (6p21.3) 63, which includes several genes associated with Notch signalling (*e.g. Notch4, FKBP5, TNXB, MTCH1* and *TNF-a*) or with stem cell maintenance and proliferation (*e.g. POU5F1, Flot1, MAPK14, Rgl2* and *Rab44*). This may point to neuronal maturation defects as a potential mechanism in fibromyalgia. c-kit, another stem cell receptor involved in neurogenesis, has been shown to regulate nociceptive sensitivity through the TRPV1 channel 65. Our findings suggest that c-kit may alter nociception at least in part by acting downstream of Notch signalling 66 to regulate the differentiation of sensory neurons.

In conclusion, we have identified a new role of Notch3 in adult neuronal differentiation and maturation, using a combination of imaging, molecular, cellular, anatomical and behavioural approaches. These lines of evidence concur to suggest that Notch3 contributes to the differentiation of adult spinal cord neural stem cells and is necessary for the maturation of lamina I–II neurons, a role distinct from its developmental function and from the action of the other Notch receptors. The impaired maturation of laminae I–II spinal cord neurons is likely to generate increased excitatory activity, resulting in a lower nociceptive threshold similar to chronic pain. Our results suggest that Notch3 knockout mice represent a new model of chronic pain and that genetic variations that interfere with neuronal differentiation, including but not limited to Notch3, may underlie human cases of intractable chronic pain or increased pain susceptibility. This neuronal differentiation-dependent model of chronic pain is fully compatible, and may overlap with synaptic plasticity or inflammation-dependent models of pain.

## Supporting Information

Figure S1 Immunofluorescence analysis of Notch3 expression in rat spinal cord, relative to neuronal marker NeuN.

Figure S2 Immunofluorescence 3D imaging shows nuclear Notch3 expression in a NeuN-stained cell (rat spinal cord), validating Notch3 expression in neurons.

Figure S3 Immunofluorescence analysis of Notch3 expression relative to oligodendrocyte markers in rat spinal cord sections.

Figure S4 Immunofluorescence analysis of Notch3 (N3, green) expression pattern in mouse spinal cord shows a similar Notch3 neuronal specificity as in rat.

Figure S5 Immunofluorescence analysis of neuronal marker synapsin I (Syn, red, arrowhead) expression in mouse dorsal horn spinal cord, after 7 days of EdU injection.

Figure S6 Immunofluorescence analysis of Notch ligands Delta and Jagged expression in rat lumbar spinal cord.

Figure S7 Comparative nuclear DAPI staining of WT and N3KO mouse spinal cord shows altered N3KO mouse morphology, similar to Fig. 6A (shown region T12-L1).

Figure S8 Immunofluorescence analysis of N3KO mouse spinal cord with neuron-specific β3 tubulin.

Figure S9 High magnification of mouse spinal cord laminae I–II (LI–II, double arrows), showing reduced NeuN staining (red) and an increased number of CR+ cells (green) in N3KO mouse relative to WT.
