# Supplementary figures and images for: Notch3 is necessary for neuronal differentiation and maturation in the adult spinal cord
Source: J Cell Mol Med. 2014 Aug 28;18(10):2103–16. doi: 10.1111/jcmm.12362 (PMC4244024; doi:10.1111/jcmm.12362)

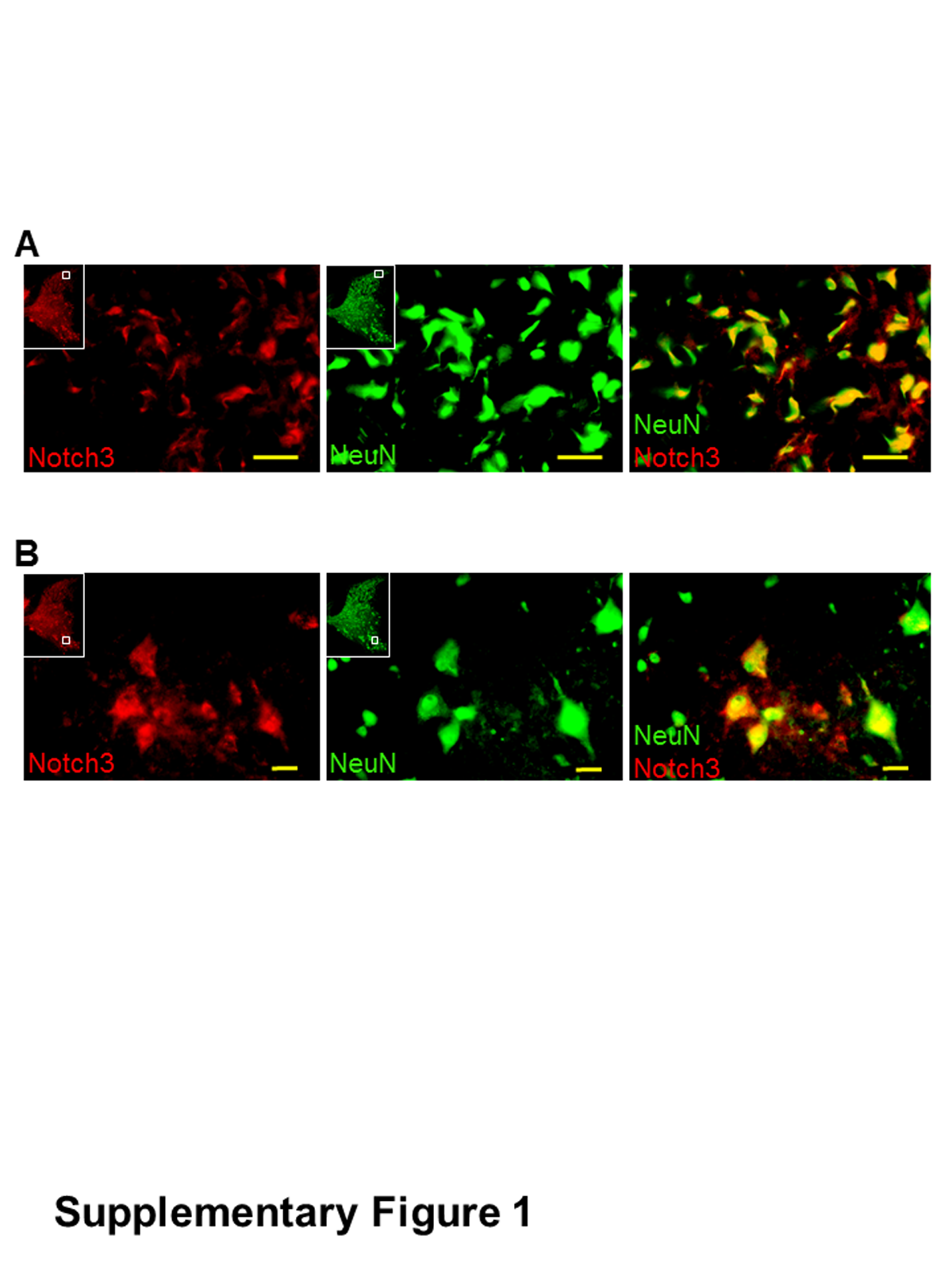

Supplement: Figure S1 — Immunofluorescence analysis of Notch3 expression in rat spinal cord, relative to neuronal marker NeuN. [file jcmm0018-2103-sd1.tif]

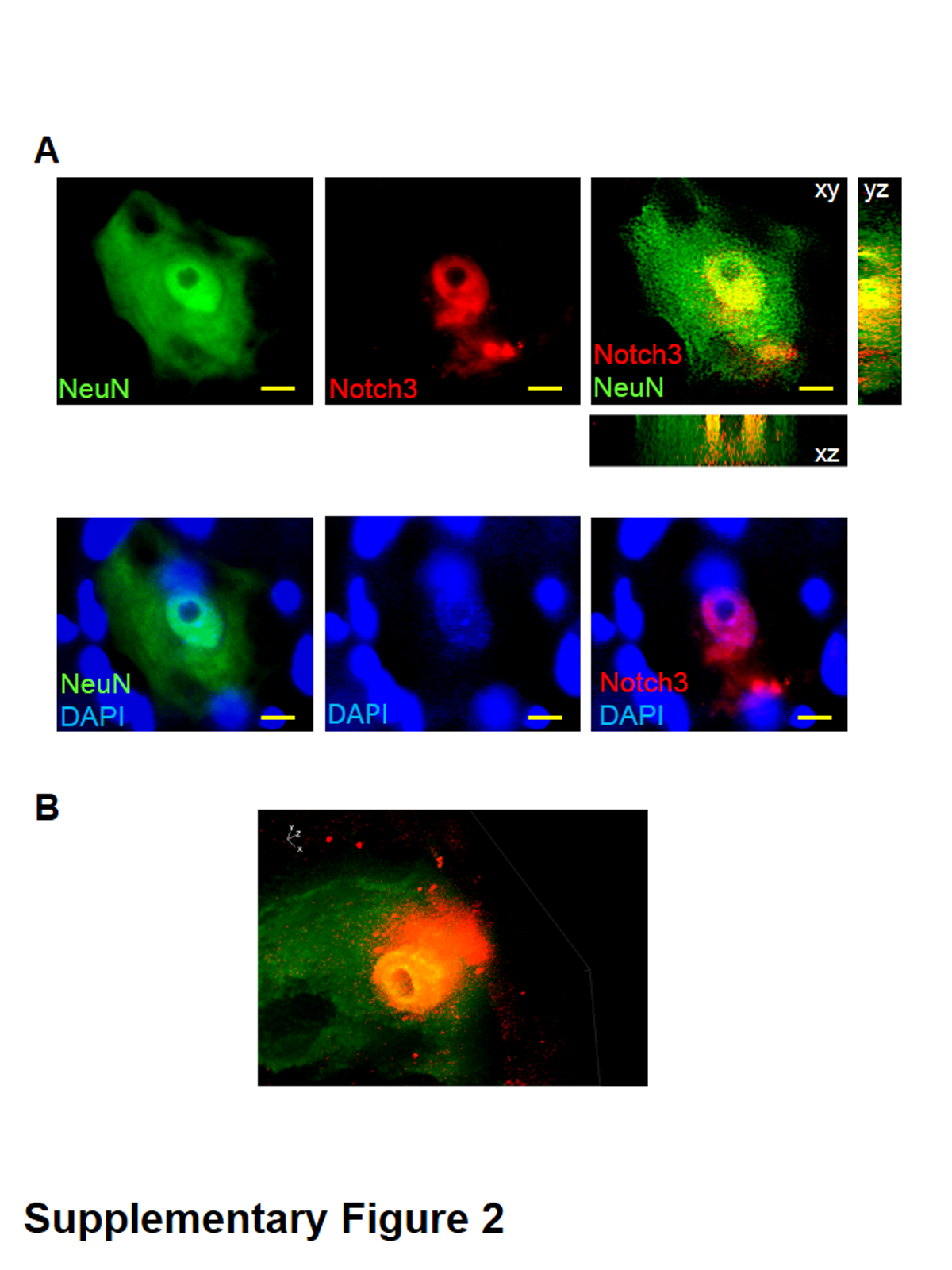

Supplement: Figure S2 — Immunofluorescence 3D imaging shows nuclear Notch3 expression in a NeuN-stained cell (rat spinal cord), validating Notch3 expression in neurons. [file jcmm0018-2103-sd2.tif]

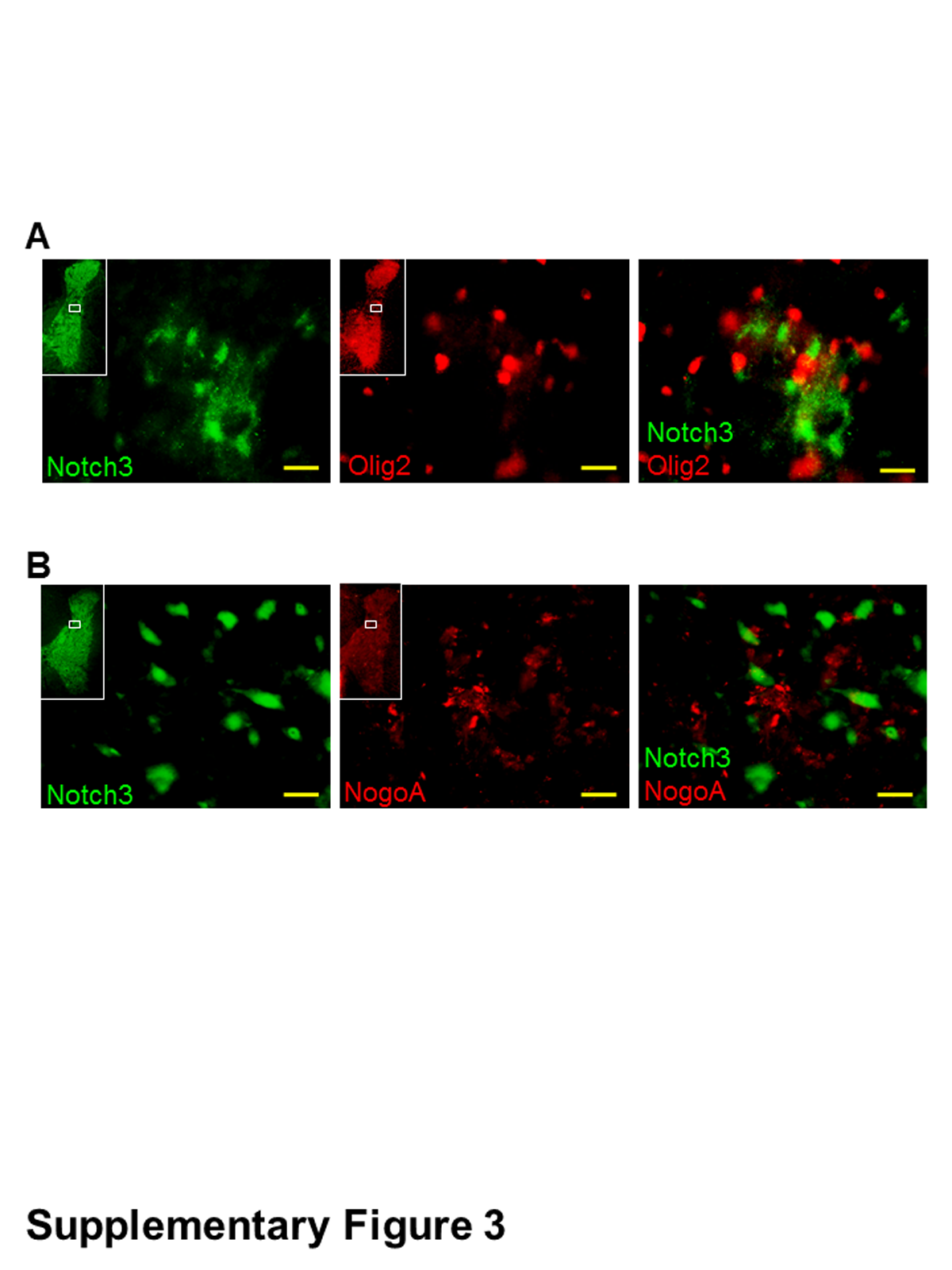

Supplement: Figure S3 — Immunofluorescence analysis of Notch3 expression relative to oligodendrocyte markers in rat spinal cord sections. [file jcmm0018-2103-sd4.tif]

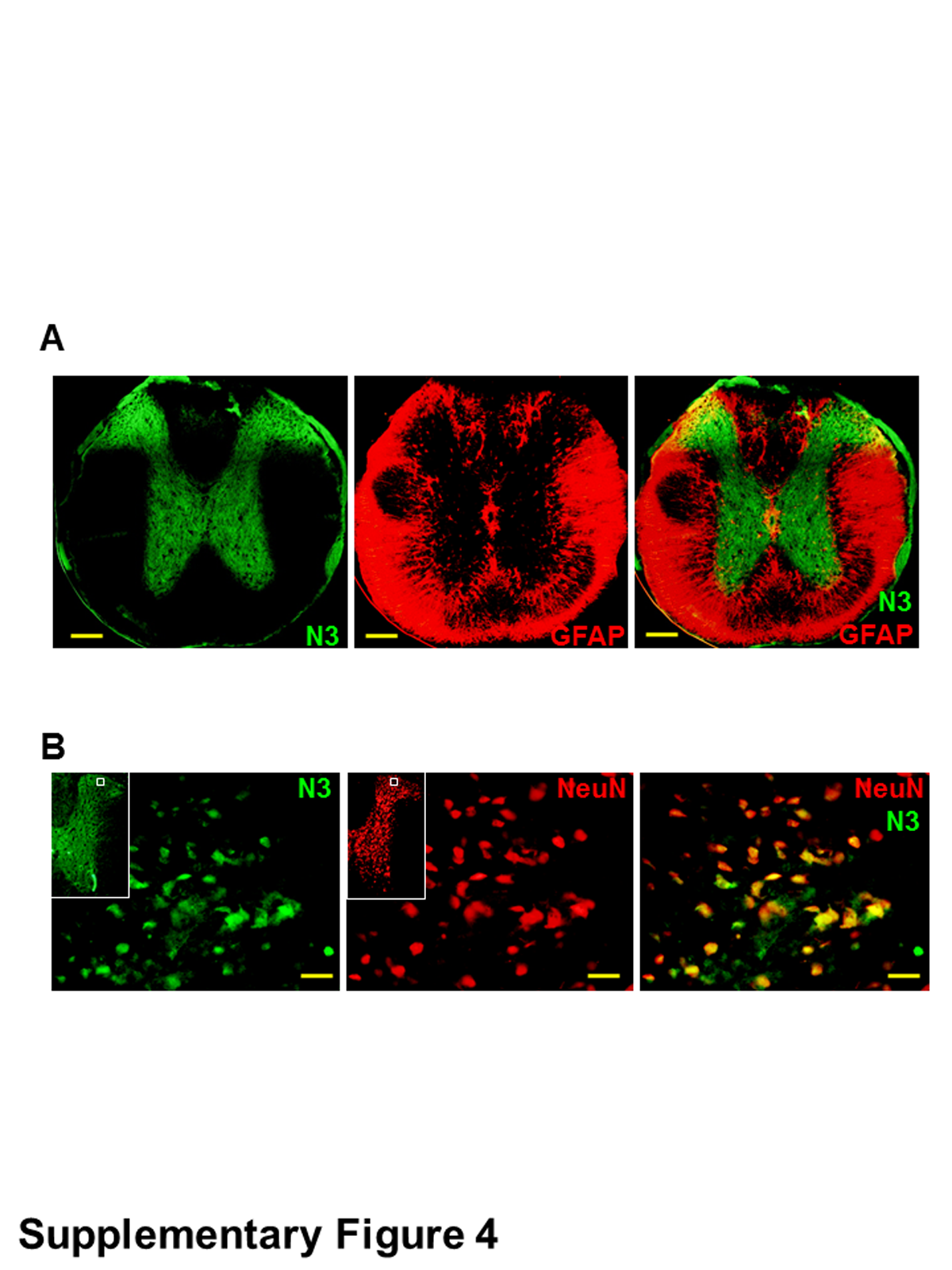

Supplement: Figure S4 — Immunofluorescence analysis of Notch3 (N3, green) expression pattern in mouse spinal cord shows a similar Notch3 neuronal specificity as in rat. [file jcmm0018-2103-sd5.tif]

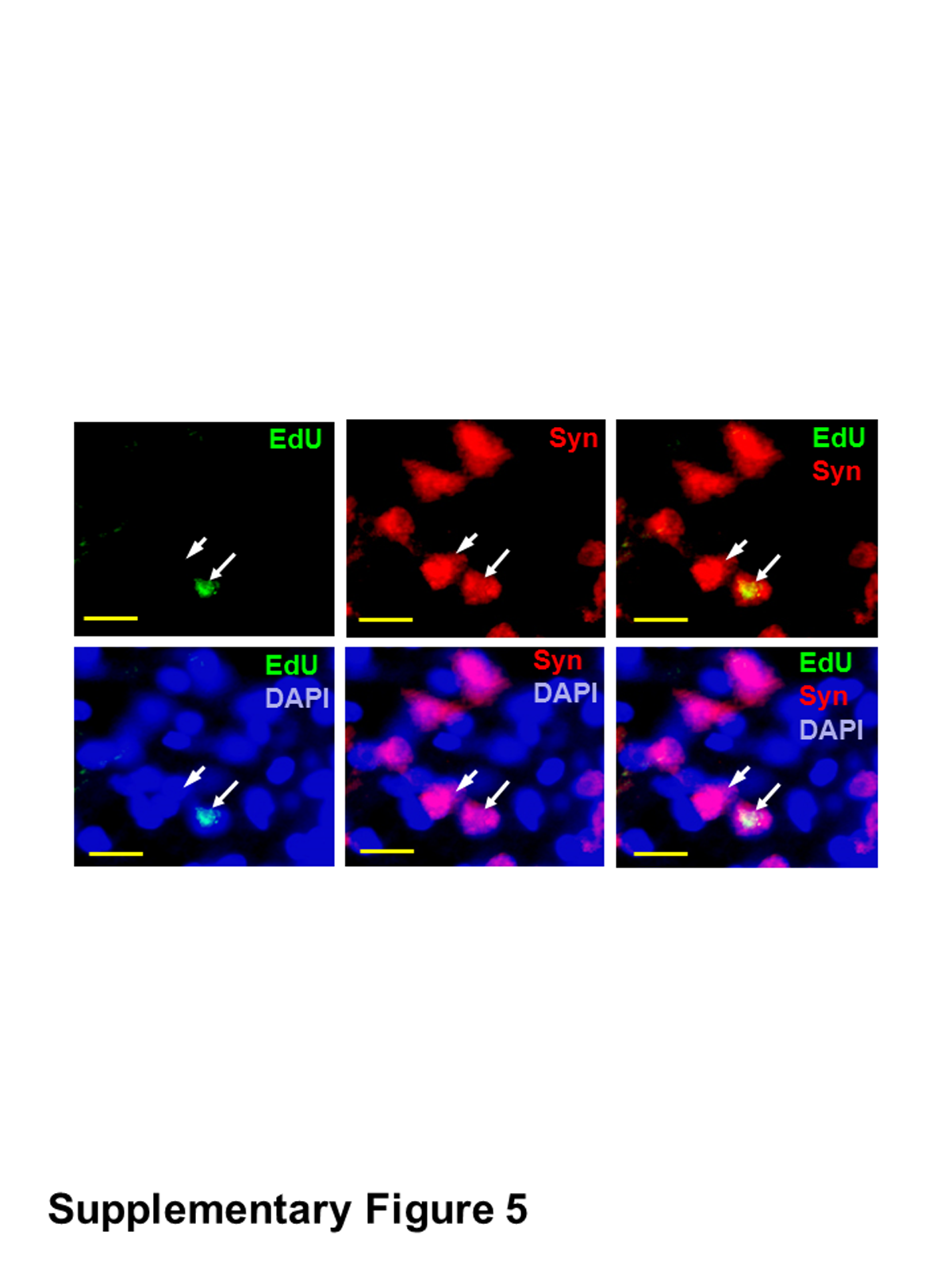

Supplement: Figure S5 — Immunofluorescence analysis of neuronal marker synapsin I (Syn, red, arrowhead) expression in mouse dorsal horn spinal cord, after 7 days of EdU injection. [file jcmm0018-2103-sd6.tif]

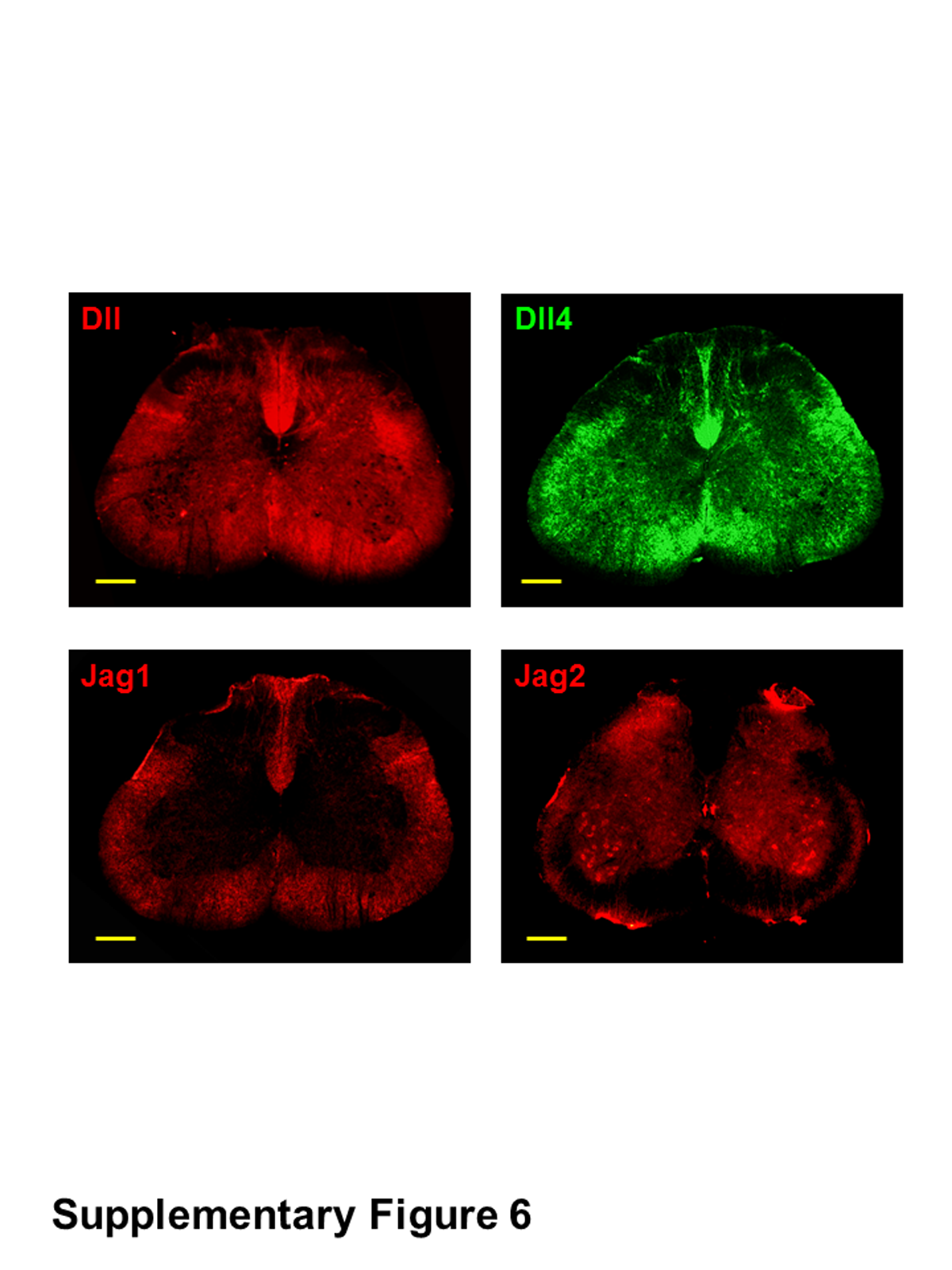

Supplement: Figure S6 — Immunofluorescence analysis of Notch ligands Delta and Jagged expression in rat lumbar spinal cord. [file jcmm0018-2103-sd7.tif]

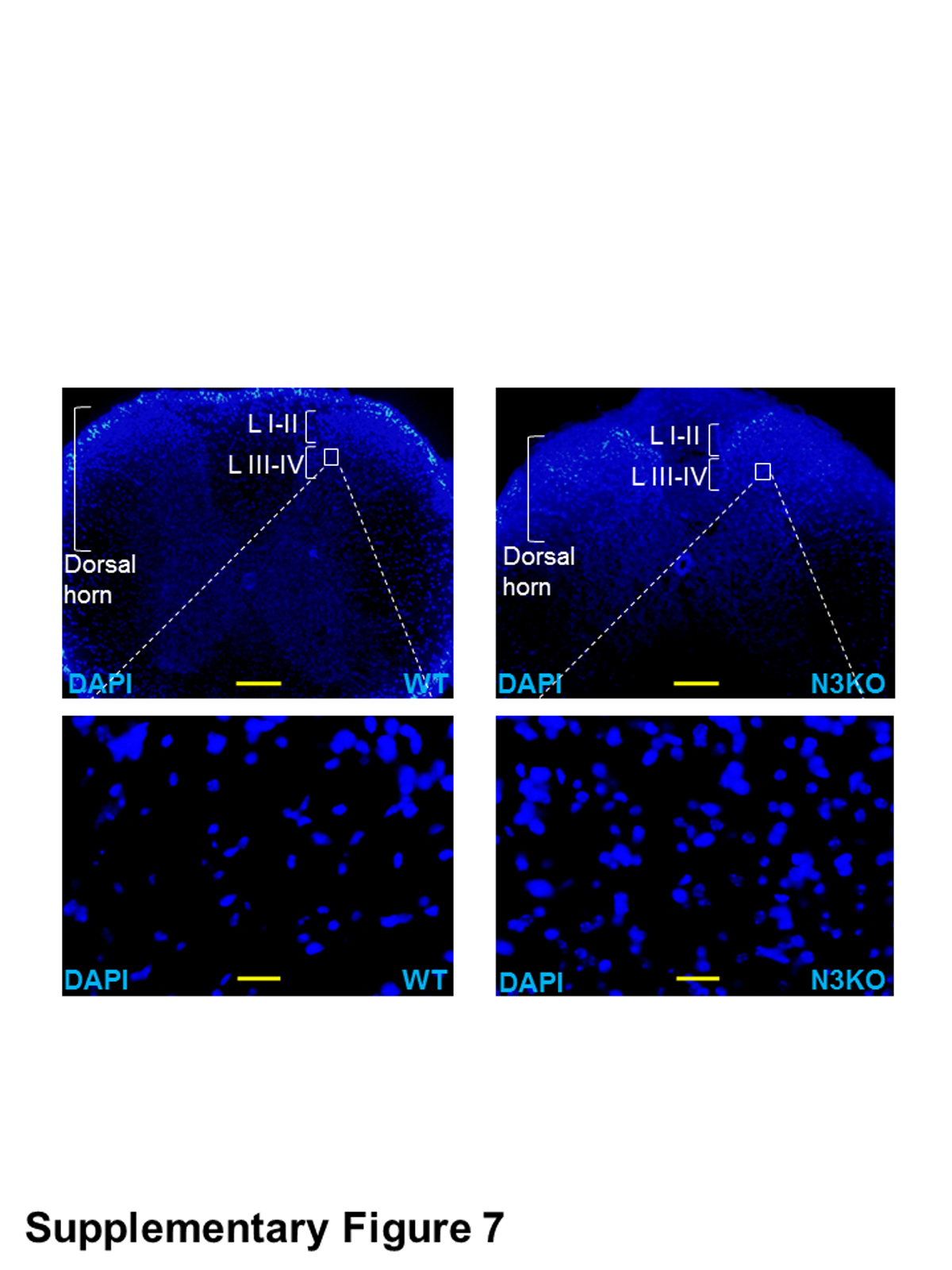

Supplement: Figure S7 — Comparative nuclear DAPI staining of WT and N3KO mouse spinal cord shows altered N3KO mouse morphology, similar to Fig. 6A (shown region T12-L1). [file jcmm0018-2103-sd8.tif]

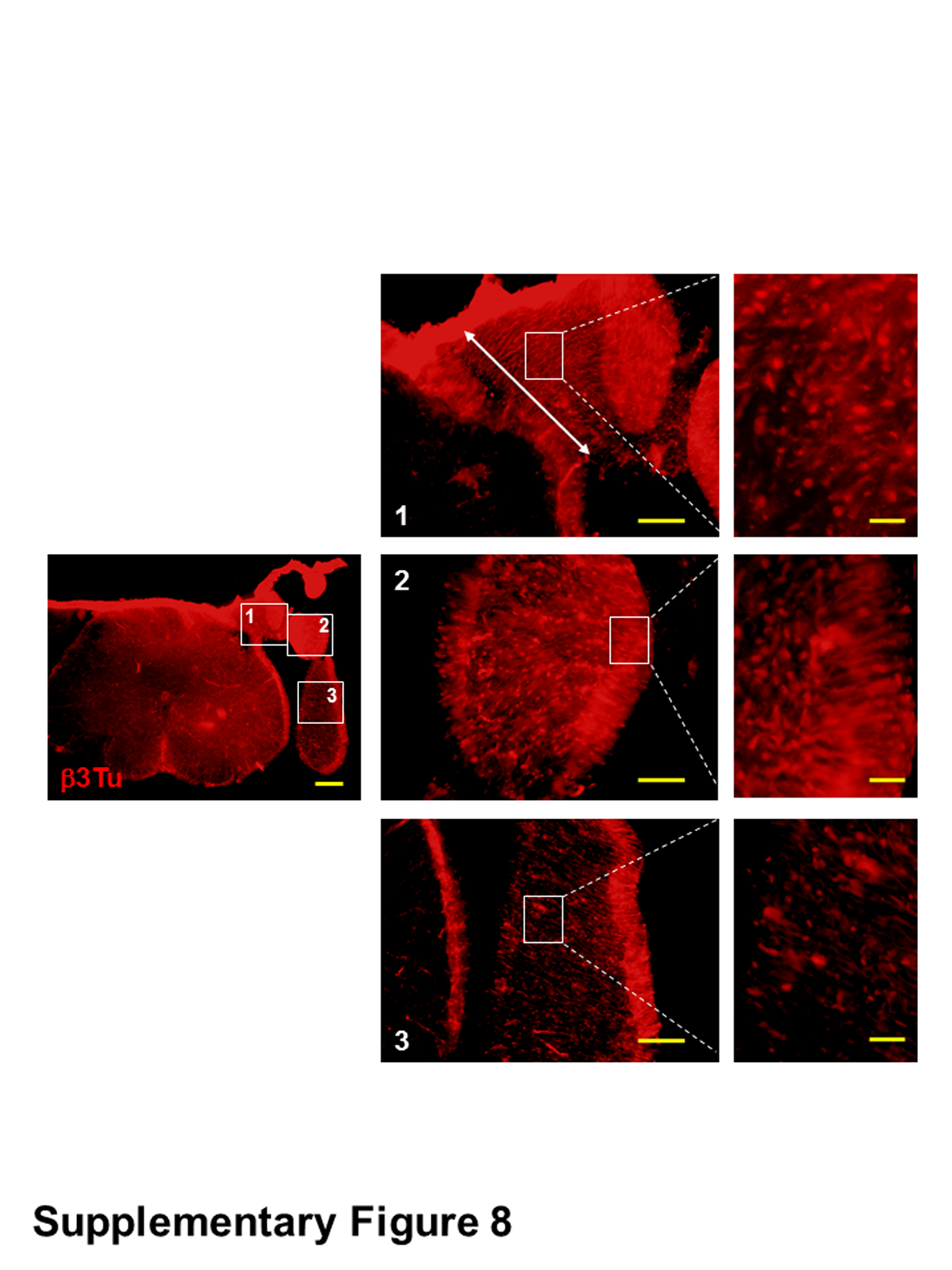

Supplement: Figure S8 — Immunofluorescence analysis of N3KO mouse spinal cord with neuron-specific β3 tubulin. [file jcmm0018-2103-sd9.tif]

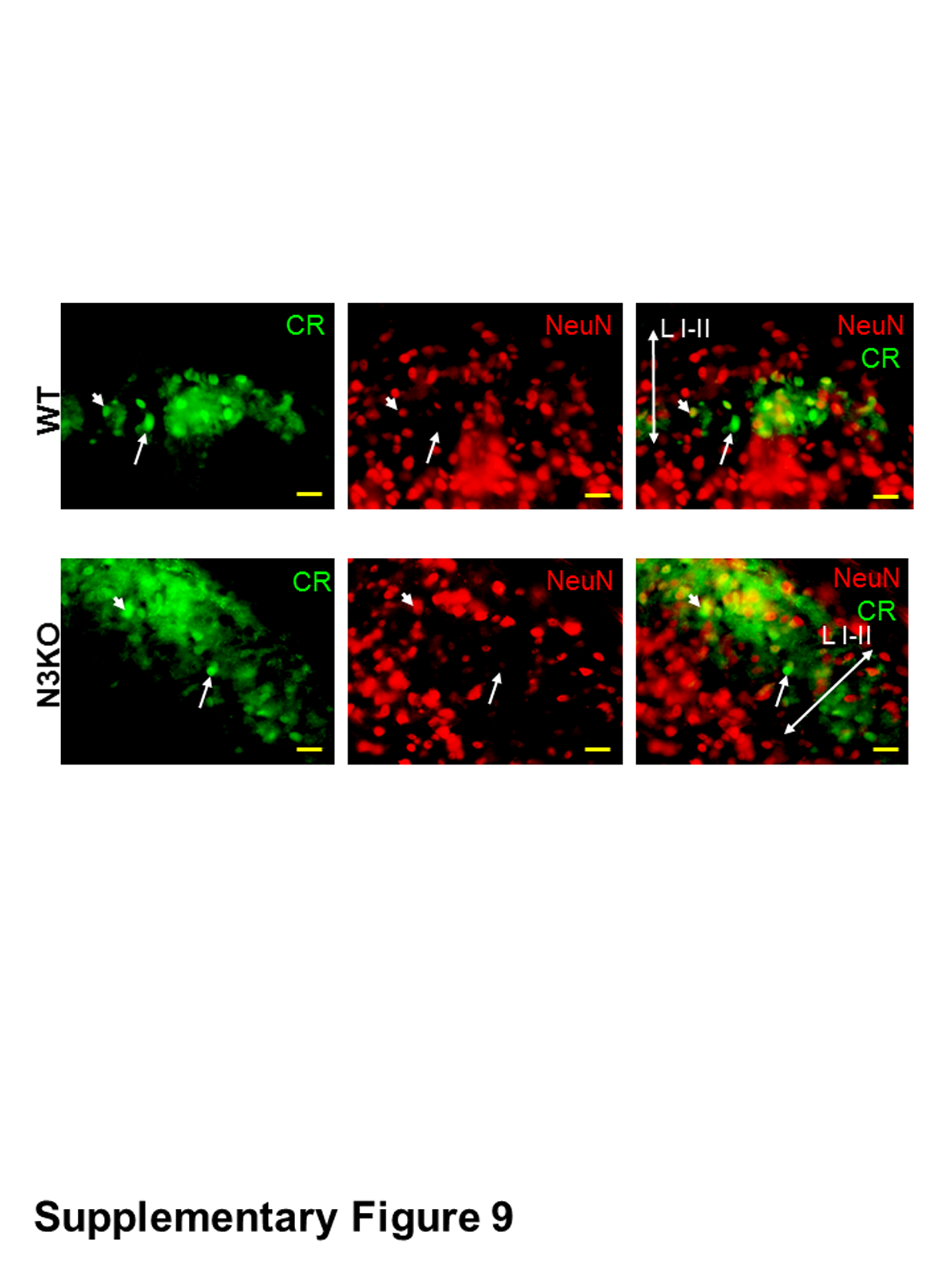

Supplement: Figure S9 — High magnification of mouse spinal cord laminae I–II (LI–II, double arrows), showing reduced NeuN staining (red) and an increased number of CR+ cells (green) in N3KO mouse relative to WT. [file jcmm0018-2103-sd10.tif]
