# Supplementary material for: Notch3 is necessary for neuronal differentiation and maturation in the adult spinal cord
Source: J Cell Mol Med. 2014 Aug 28;18(10):2103–16. doi: 10.1111/jcmm.12362 (PMC4244024; doi:10.1111/jcmm.12362)
Supplement: Supplementary file 11 [file jcmm0018-2103-sd11.docx]

**Supplementary Figure Legends**

**Supplementary Figure 1.** Immunofluorescence analysis of Notch3 expression in rat spinal cord, relative to neuronal marker NeuN. Notch3 is expressed throughout the spinal cord grey matter, including in small neurons in laminae I-II (**A**) and in some large ventral horn motor neurons (**B**). Scale bars: 20 μm (A), 50 μm (B).

**Supplementary Figure 2**. Immunofluorescence 3D imaging shows nuclear Notch3 expression in a NeuN-stained cell (rat spinal cord), validating Notch3 expression in neurons. **A**, Notch3 (Cy3, red) and NeuN (FITC, green) are co-expressed in a cell nucleus. Right panel also shows the xz and yz projections of a 3D reconstruction of the cell with a 0.5 μm increment, confirming the co-localization of Notch3 and NeuN staining in the nucleus. All cells present are indicated by nuclear DAPI staining (blue). Scale bars: 5 μm. **B**, 3D animation of same cell as in A, showing that the Notch3-stained nucleus is positioned within the NeuN-stained cell.

**Supplementary Figure 3**. Immunofluorescence analysis of Notch3 expression relative to oligodendrocyte markers in rat spinal cord sections. **A**, Notch3 shows no co-localization with Olig2, a marker for oligodendrocyte precursors. **B**, Notch3 does not co-localize with NogoA, a marker for mature oligodendrocytes. Scale bars: 20 μm.

**Supplementary Figure 4.** Immunofluorescence analysis of Notch3 (N3, green) expression pattern in mouse spinal cord shows a similar Notch3 neuronal specificity as in rat. Mouse Notch3 expression is complementary to GFAP (**A**, compare to Fig. 1A) and colocalizes with NeuN (**B**, compare with Fig. 1B). Scale bars: 200 μm.

**Supplementary Figure 5.** Immunofluorescence analysis of neuronal marker synapsin I (Syn, red, arrowhead) expression in mouse dorsal horn spinal cord, after 7 days of EdU injection. The same spinal cords from EdU-treated mice have been used for synapsin I and Notch3 detection (Fig. 3B). Some Syn+ cells show EdU (green, arrow) nuclear staining, indicative of newly produced neuron progenitors. Nuclear DAPI staining (blue) indicates all cells present. Scale bars: 10 μm.

**Supplementary Figure 6.** Immunofluorescence analysis of Notch ligands Delta and Jagged expression in rat lumbar spinal cord. Delta (Dll, red), Delta4 (Dll4, green) and Jagged1 (Jag1, red) are expressed preferentially in the white matter, while Jagged2 (Jag2, red) is expressed mostly in the grey matter, similar to Notch3. Some Jag2-stained neurons are visible even at low magnification. Scale bars: 200 μm.

**Supplementary Figure 7.** Comparative nuclear DAPI staining of WT and N3KO mouse spinal cord shows altered N3KO mouse morphology, similar to Fig. 6A (shown region T12-L1). DAPI is a general nuclear stain and accurately reflects spinal cord morphology, circumventing potential bias that may result from selective imaging with neuronal markers such as NeuN and CR. N3KO mice have shorter dorsal horns (81% + 7%, n=5, **p*<0.05), and a higher cell density in laminae III-IV (136% + 9%, n=5, ***p*<0.01), compared to WT mice. Scale bars: 200 μm, 20 μm (details).

**Supplementary Figure 8.** Immunofluorescence analysis of N3KO mouse spinal cord with neuron-specific β3 tubulin. Details show a short, broad (panel 1, double arrow) spinal nerve, with inclusion of numerous small neurons, followed by a dorsal root ganglion with a high density of neurons (panel 2) and a dorsal root ganglion with a lower neuronal density (panel 3), similar to the morphology revealed by NeuN staining (see Fig. 6B). Scale bars: 200 μm, 100 μm (panels 1-3), 20 μm (details).

**Supplementary Figure 9**. High magnification of mouse spinal cord laminae I-II (LI-II, double arrows), showing reduced NeuN staining (red) and an increased number of CR+ cells (green) in N3KO mouse relative to WT. In both WT and N3KO mice, some CR+ cells show various degrees of overlap with NeuN staining (arrowheads), however some CR+ cells do not express NeuN (arrows), potentially indicating immature neurons. Scale bars: 20 μm.
